# Supplementary material for: The genome and transcriptome of the snail Biomphalaria sudanica s.l.: immune gene diversification and highly polymorphic genomic regions in an important African vector of Schistosoma mansoni
Source: BMC Genomics. 2024 Feb 19;25:192. doi: 10.1186/s12864-024-10103-w (PMC10875847; doi:10.1186/s12864-024-10103-w)

Supplementary Figure 1

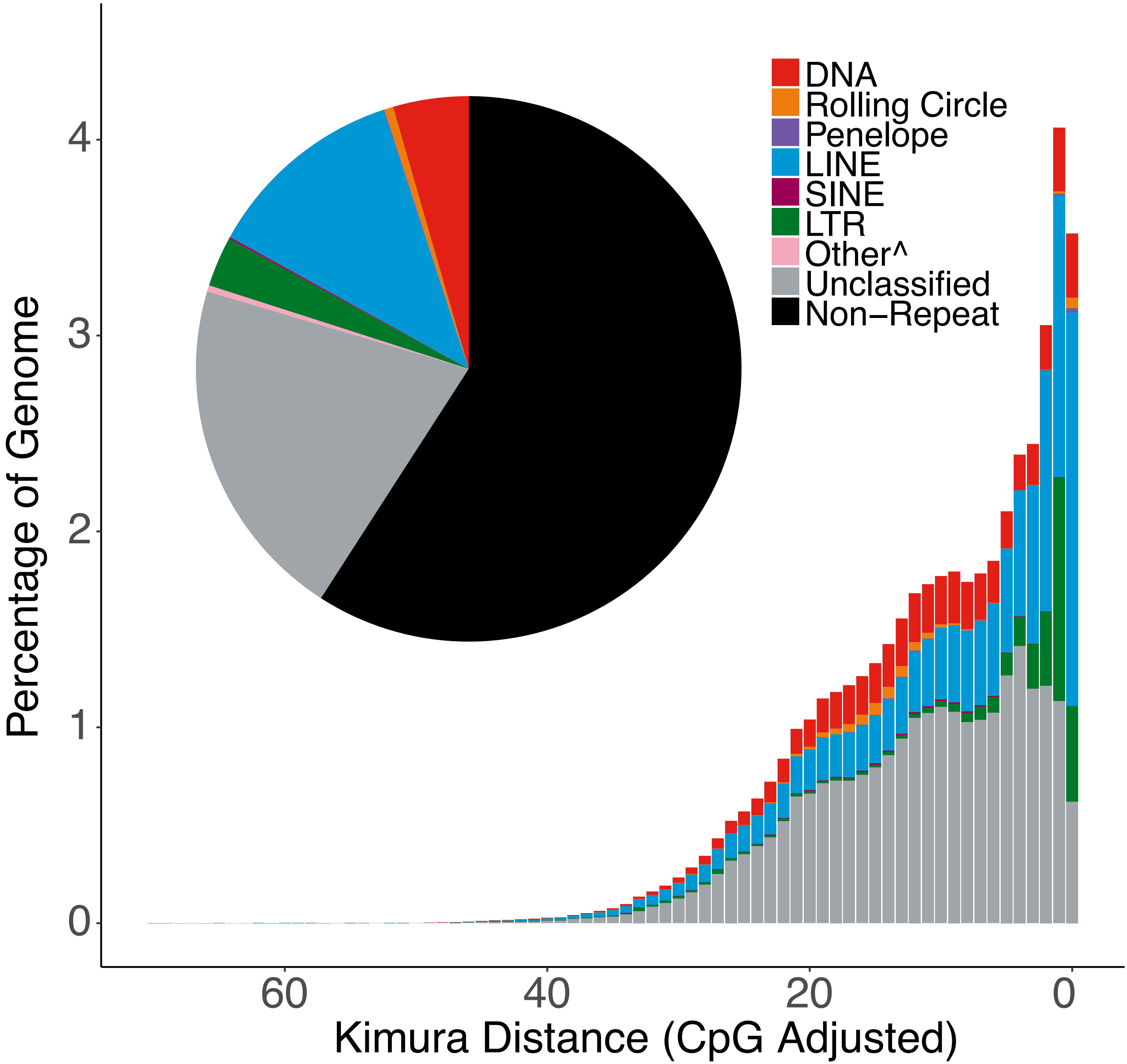

Supplementary Figure 2

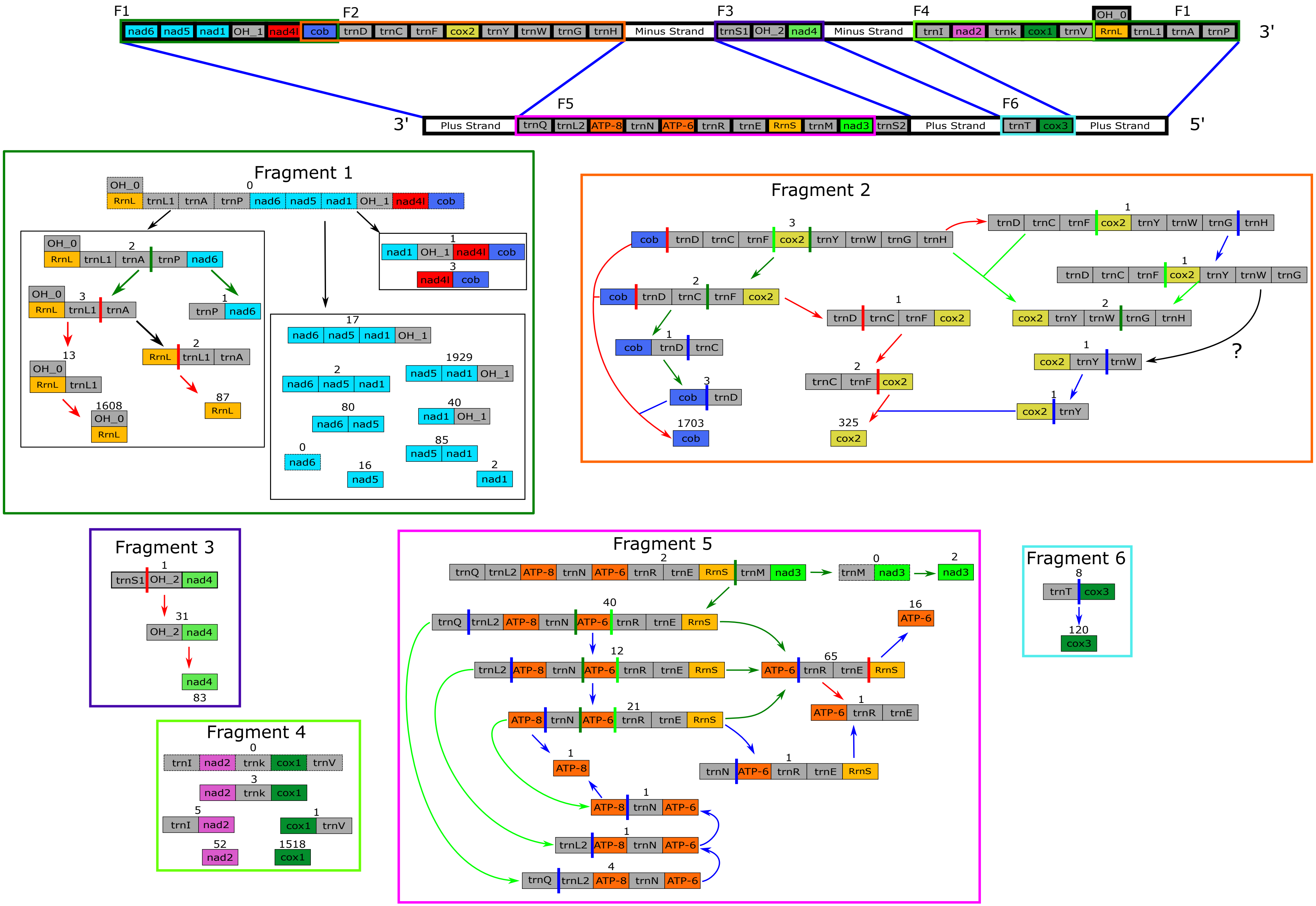

Supplementary Figure 3

A. Linkage Group 6

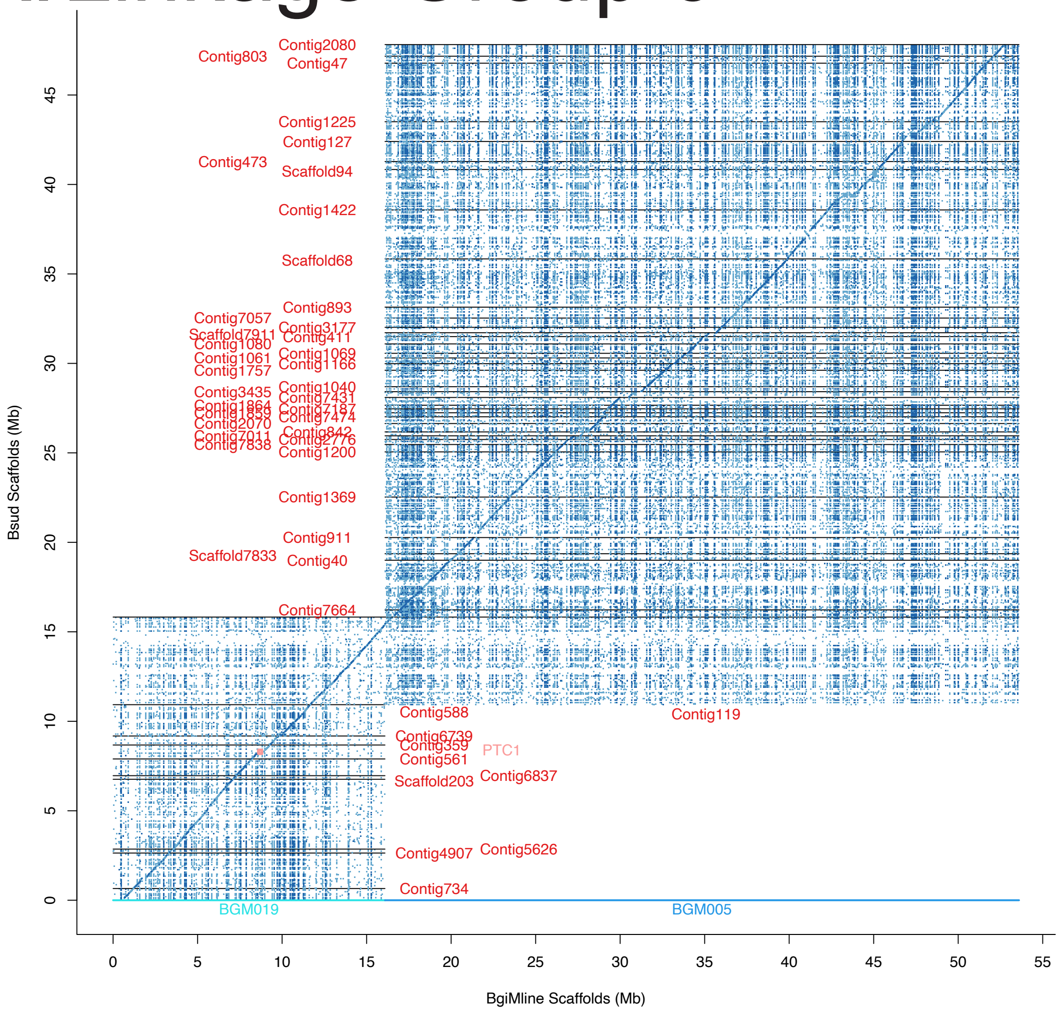

B. Linkage Group 10

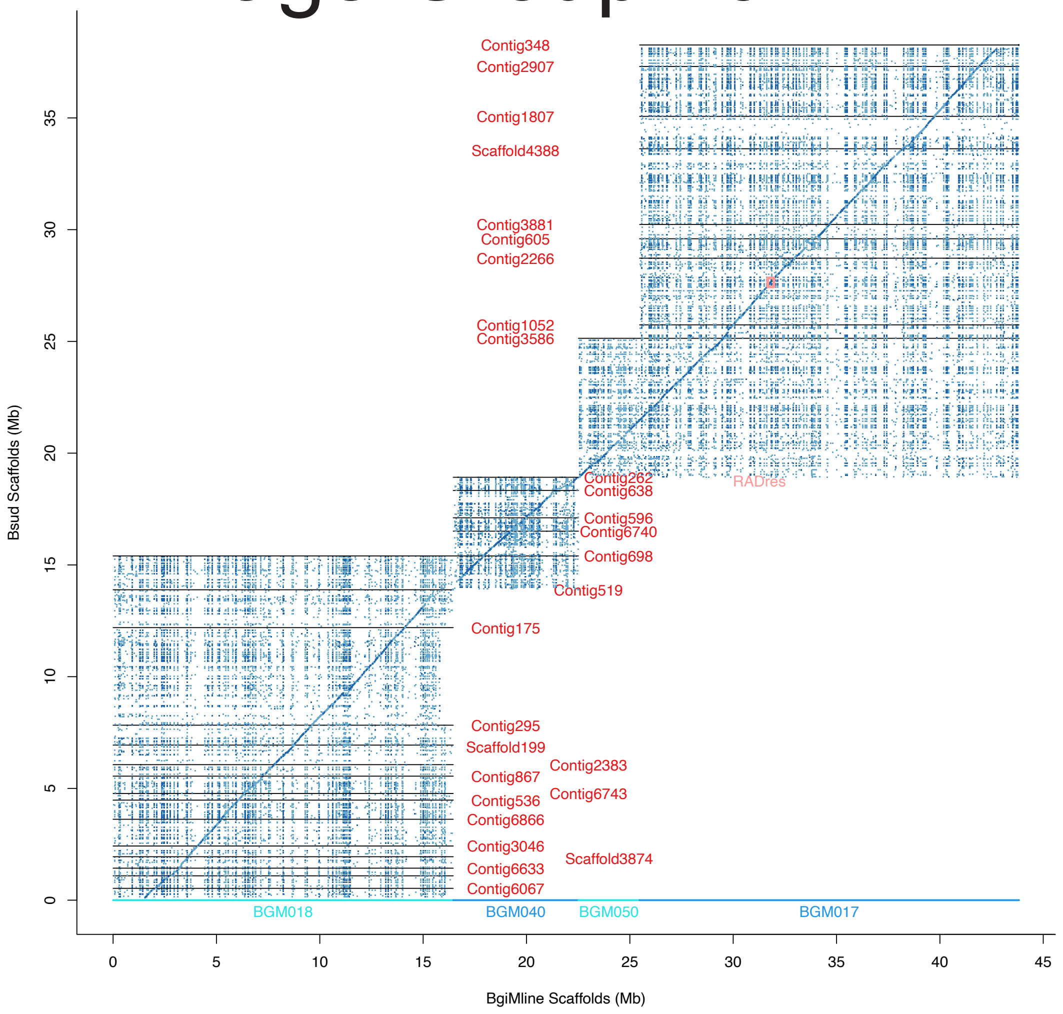

C. Linkage Group 16

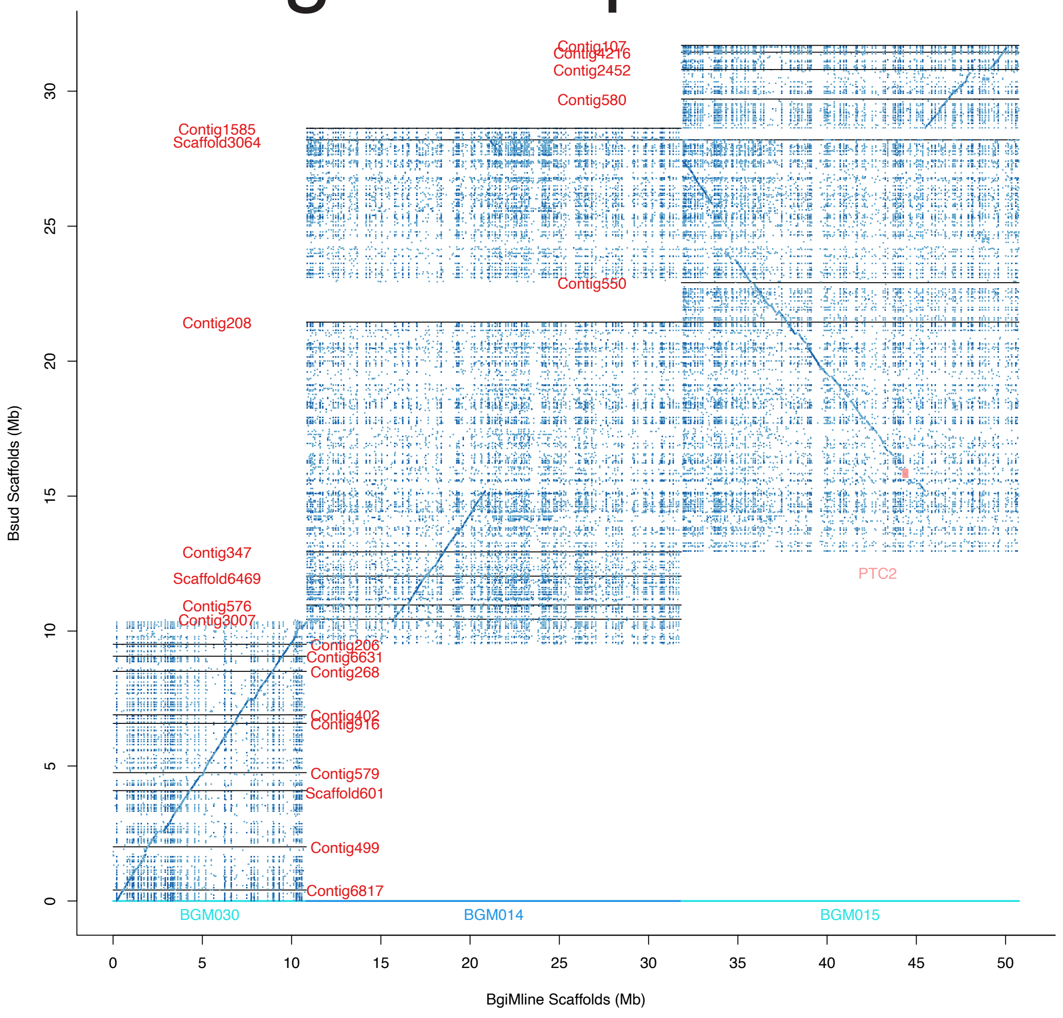

Supplementary Figure 4

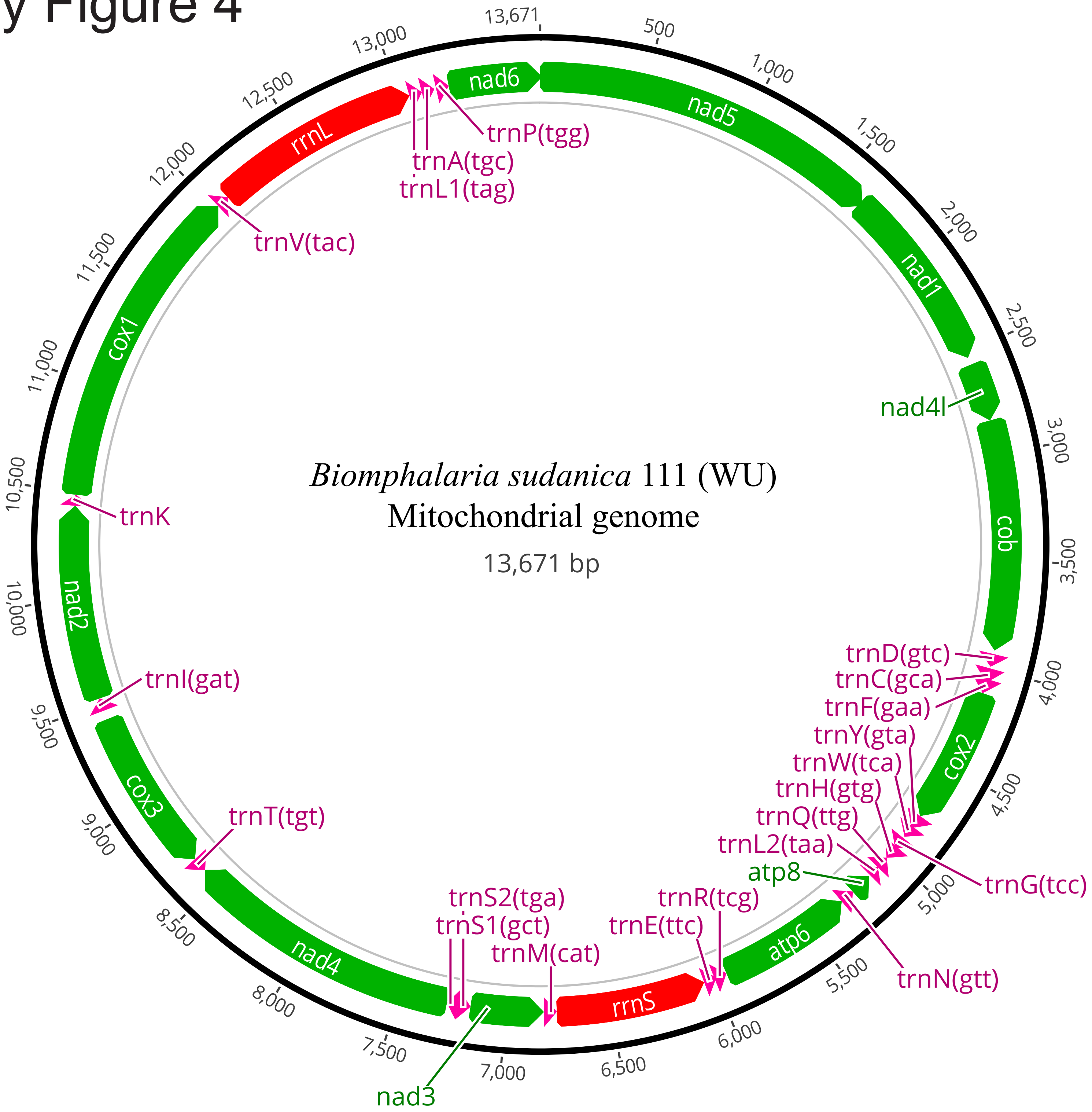

Supplementary Figure 5

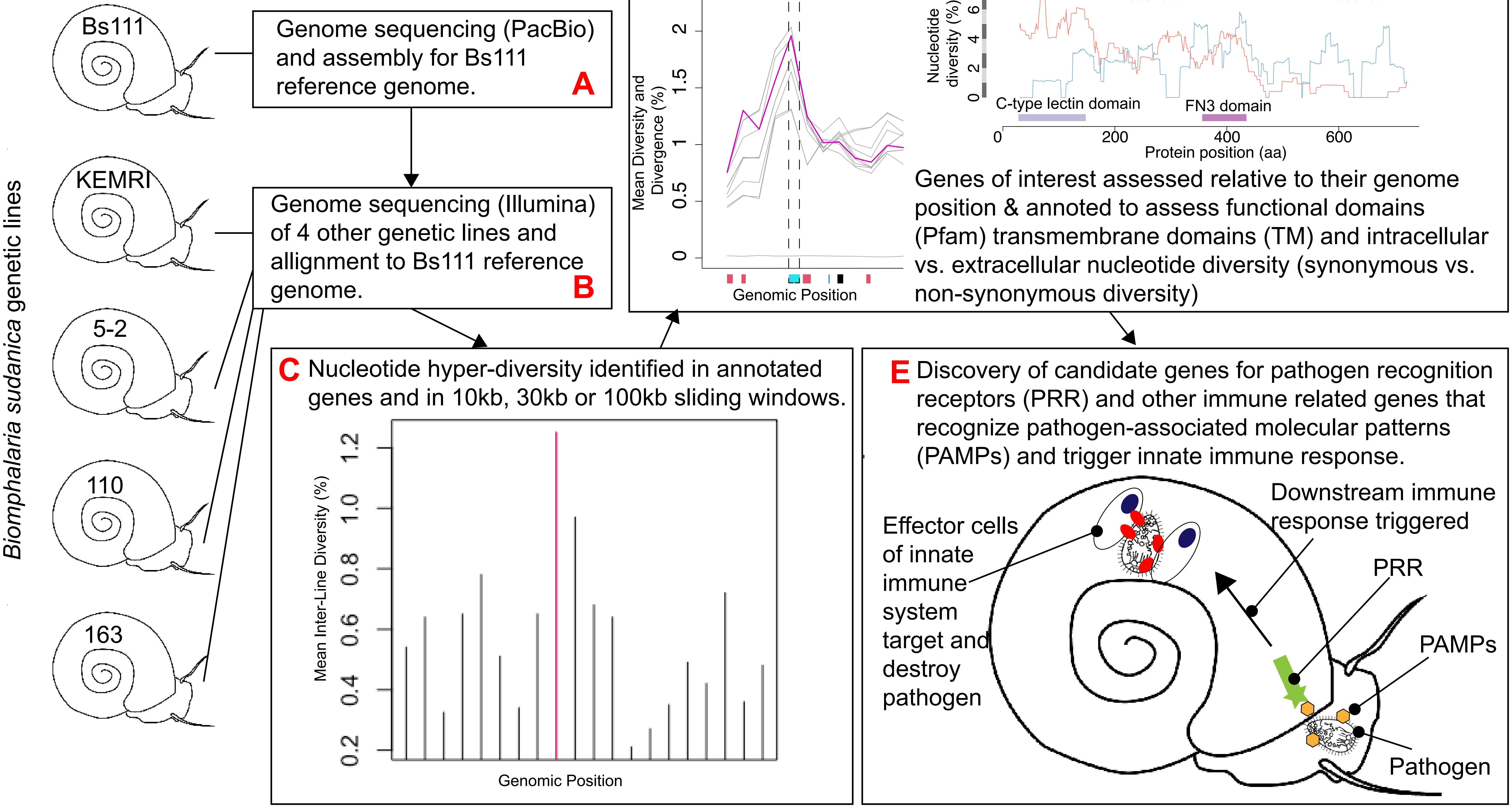

Supplement: Supplementary file 1 — Additional file 1: Supplementary Figure 1. Repetitive elements in the Biomphalaria sudanica genome. ^ “Other” indicates a Simple Repeat, Microsatellite, RNA. Supplementary Figure 2. Schematic presentation of the mitochondrial gene trimming process for the transcripts, showing regions of primary transcription (Fragment 1-6) on the plus and minus strand, and the trimming processes of these primary transcripts into pre-mRNA. Numbers above transcripts represent the RNA sequence depth from aligned PacBio IsoSeq ccs data. Supplementary Figure 3. Dot plots of Biomphalaria sudanica linkage groups 6 (A), 10 (B) and 16 (C), composed of multiple scaffolds determined using the B. glabrata iM line linkage map (Bu et al., 2022). Dots represent 600bp segments; dark blue is ≥97.5% sequence similarity, light blue is ≥90% sequence similarity. Supplementary Figure 4. Mitochondrial genome of Biomphalaria sudanica with point of origin set to the start of the nad5 gene. Supplementary Figure 5. Schematic overview of the methods employed to delimit pathogen recognition receptors (PRR) and other immune genes of Biomphalaria sudanica under balancing selection, determined through the analysis of high intraspecific genetic diversity regions, potentially relevant to the resistance and susceptibility of this species to Schistosoma mansoni. [file 12864_2024_10103_MOESM1_ESM.pdf]
