# Supplementary material for: The genome and transcriptome of the snail Biomphalaria sudanica s.l.: immune gene diversification and highly polymorphic genomic regions in an important African vector of Schistosoma mansoni
Source: BMC Genomics. 2024 Feb 19;25:192. doi: 10.1186/s12864-024-10103-w (PMC10875847; doi:10.1186/s12864-024-10103-w)

Species tree generated in Orthofinder using the Species Tree of All Genes (STAG) algorithm, as shown in Figure 1 on the main text. Node support values represent the bipartition proportions in each of the individual species tree estimates. Branch lengths (rounded up to 3d.p.) represent the average number of substitutions per site across all the individual trees inferred from each gene family. The number of (significant/total) gene families expanded (blue) and contracted (red) of Biomphalaria species, and outgroups Elysia marginata and Bulinus truncatus as determined in Café are shown for each internal and terminal node (<0> to <13>).

The following pages show node by node the REVIGO summaries of the GO terms enriched among the protein families with significant Expansion and Contractions. Results for the outgroup species and nodes are not included.

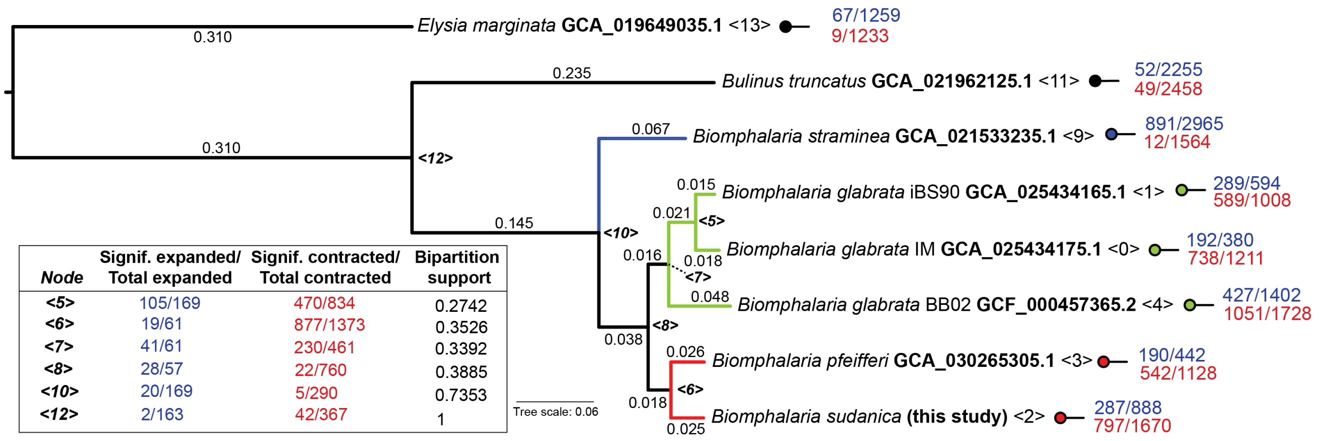

Node	Signif. expanded/ Total expanded	Signif. contracted/ Total contracted	Bipartition support
<5>	105/169	470/834	0.2742
<6>	19/61	877/1373	0.3526
<7>	41/61	230/461	0.3392
<8>	28/57	22/760	0.3885
<10>	20/169	5/290	0.7353
<12>	2/163	42/367	1

Nodes with REVIGO results:
Node 0: Species *B. glabrata*, strain IM (GCA_025434175.1).
Node 1: Species *B. glabrata*, strain iBS90 (GCA_025434165.1).
Node 2: Species *B. sudanica* (this study).
Node 3: Species *B. pfeifferi* (GCA_030265305.1).
Node 4: Species *B. glabrata*, strain BB02 (GCF_000457362.2).
Node 5: Common ancestor between *B. glabrata* strains iBS90 and IM.
Node 6: Common ancestor between the African Biomphalaria species.
Node 7: Common ancestor between the three *B. glabrata* strains.
Node 8: Common ancestor between the three *B. glabrata* strains and the African species.
Node 9: Species *B. straminea* (GCF_021533235.1).
Node 10: Common ancestor of all Biomphalaria species.

Node 0: *Biomphalaria glabrata* IM GCA_025434175.1
Protein Family Expansion

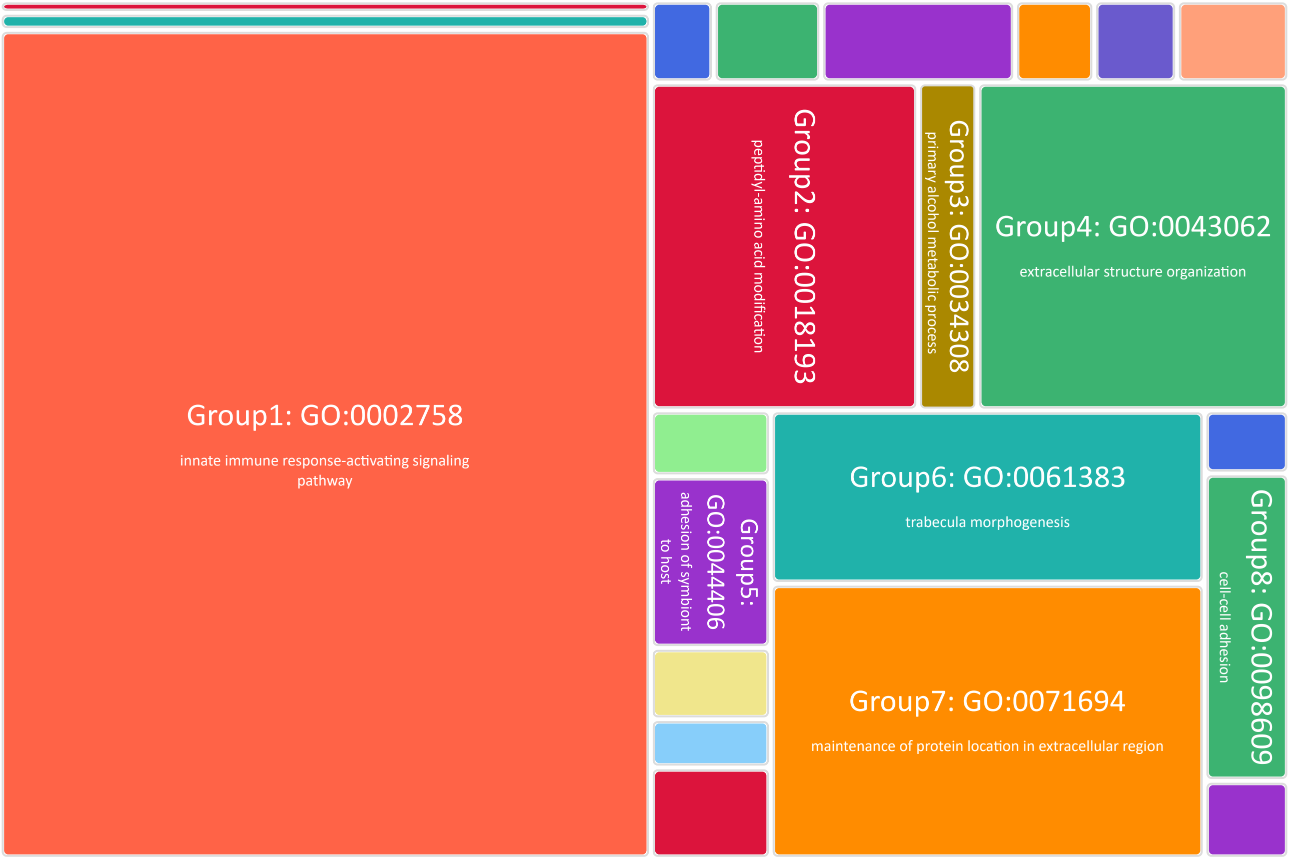

Node 0: *Biomphalaria glabrata* IM GCA_025434175.1
Protein Family Contraction

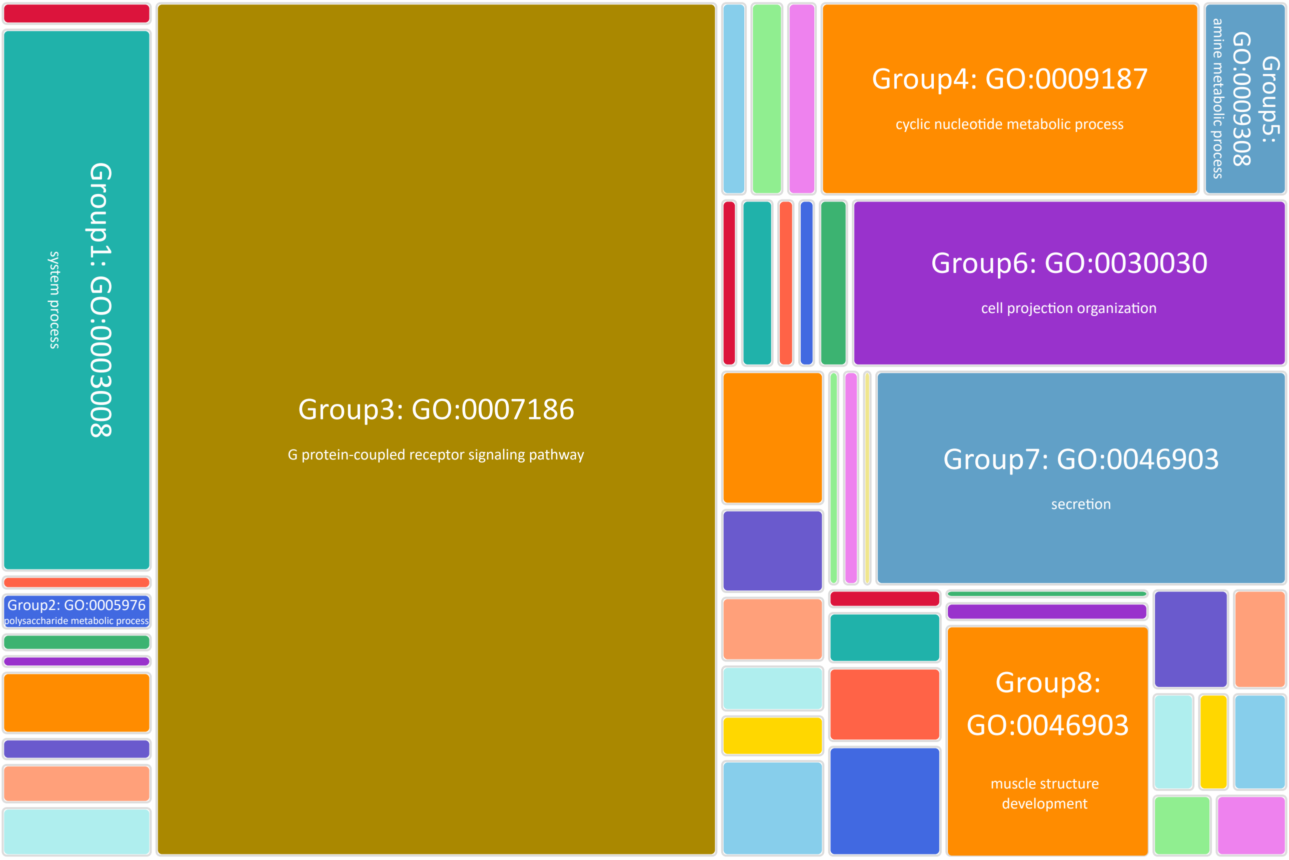

Node 1: *Biomphalaria glabrata* iBS90 GCA_025434165.1
Protein Family Expansion

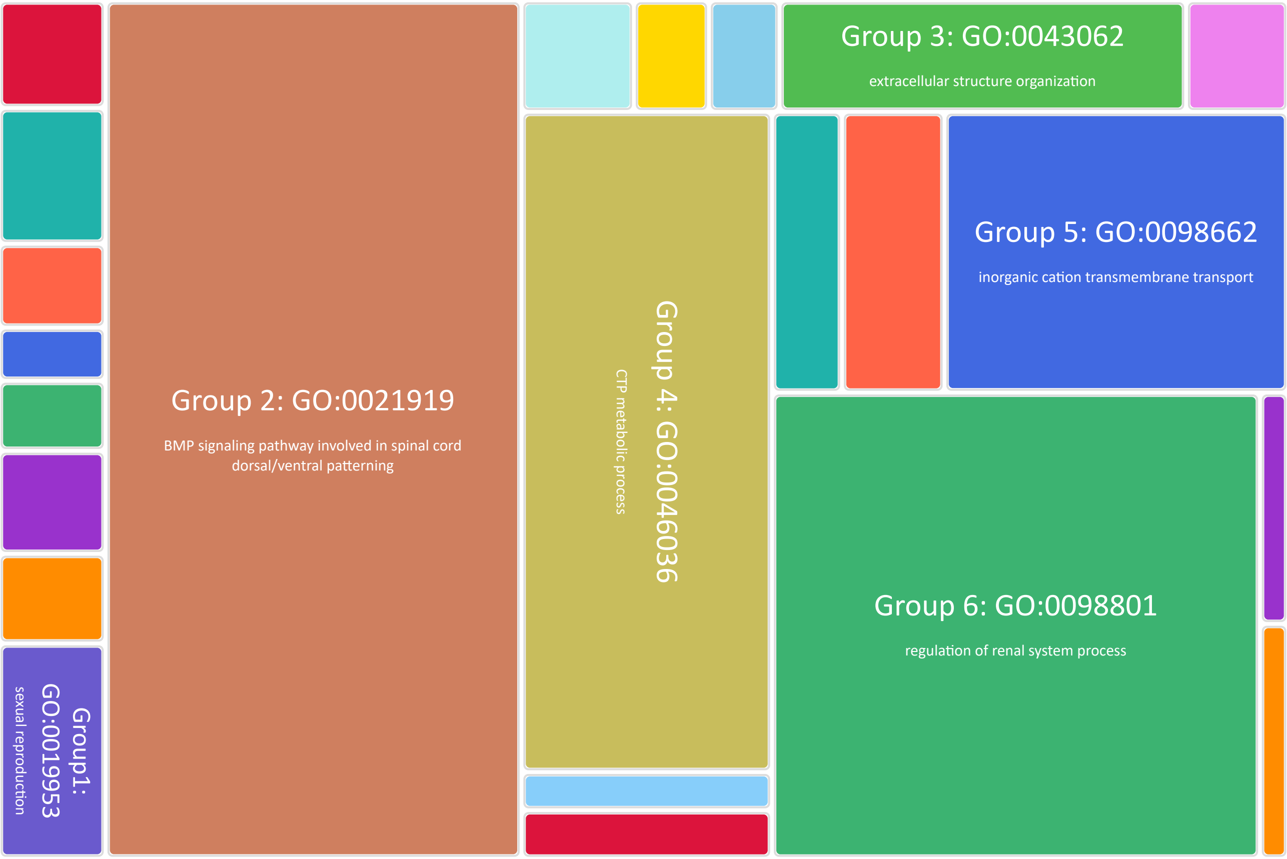

Node 1: *Biomphalaria glabrata* iBS90 GCA_025434165.1
Protein Family Contraction

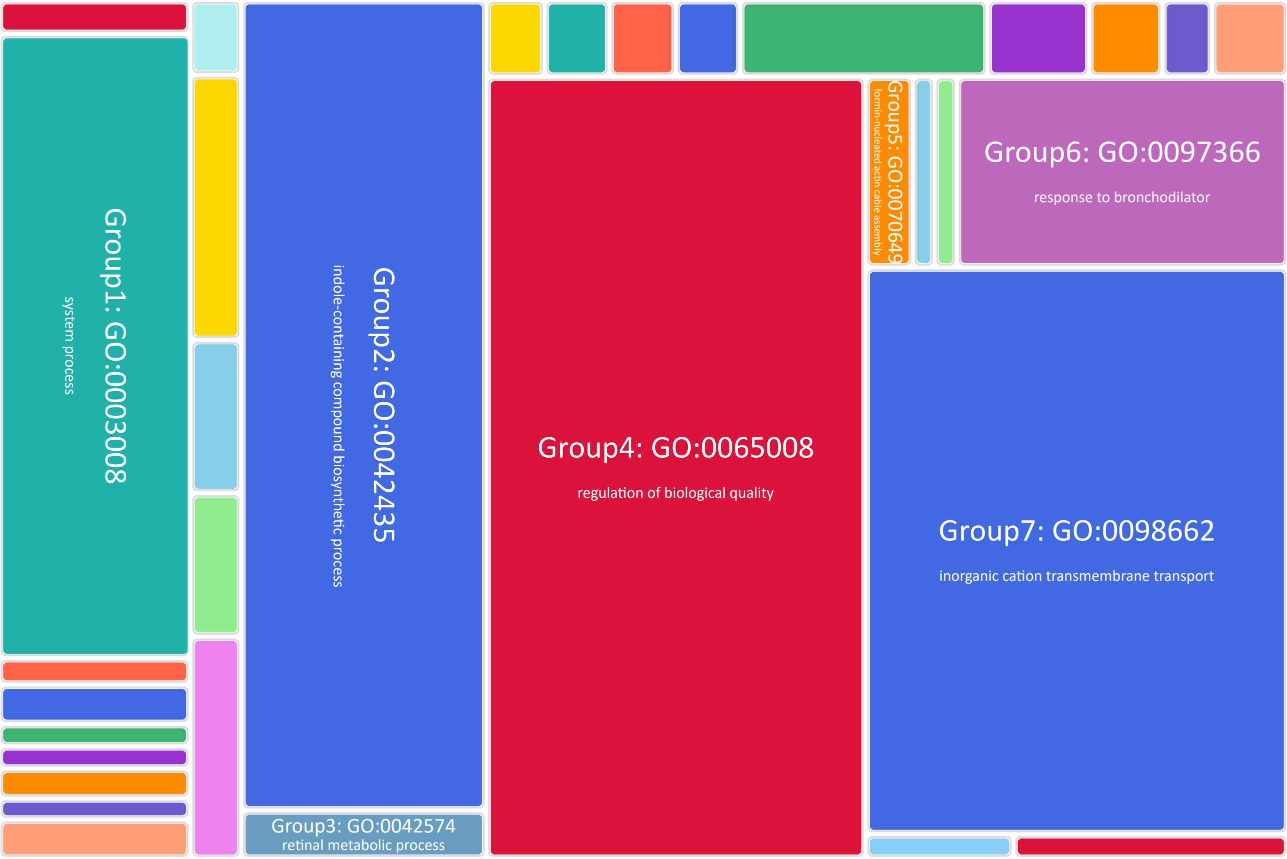

Node 2: *Biomphalaria sudanica*
Protein Family Expansion

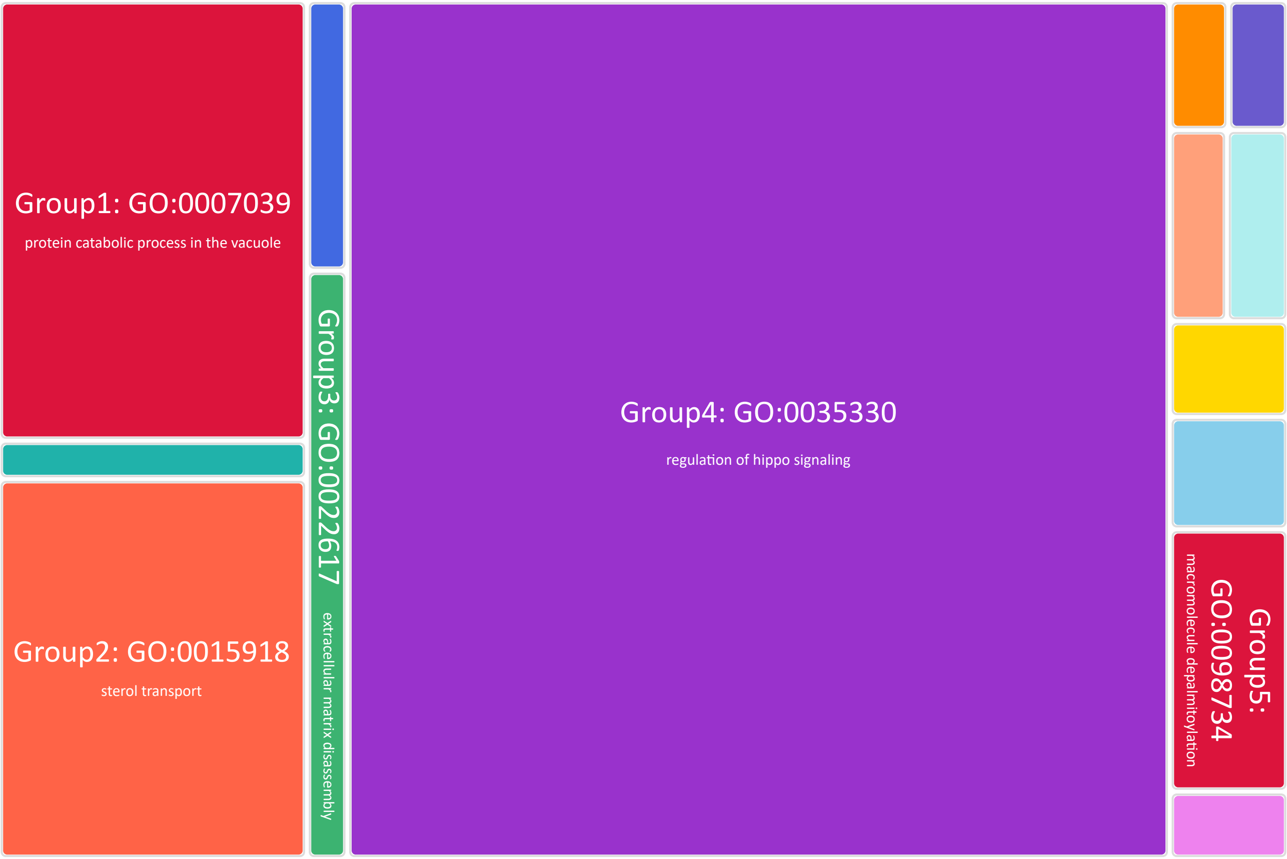

Node 2: *Biomphalaria sudanica*
Protein Family Contraction

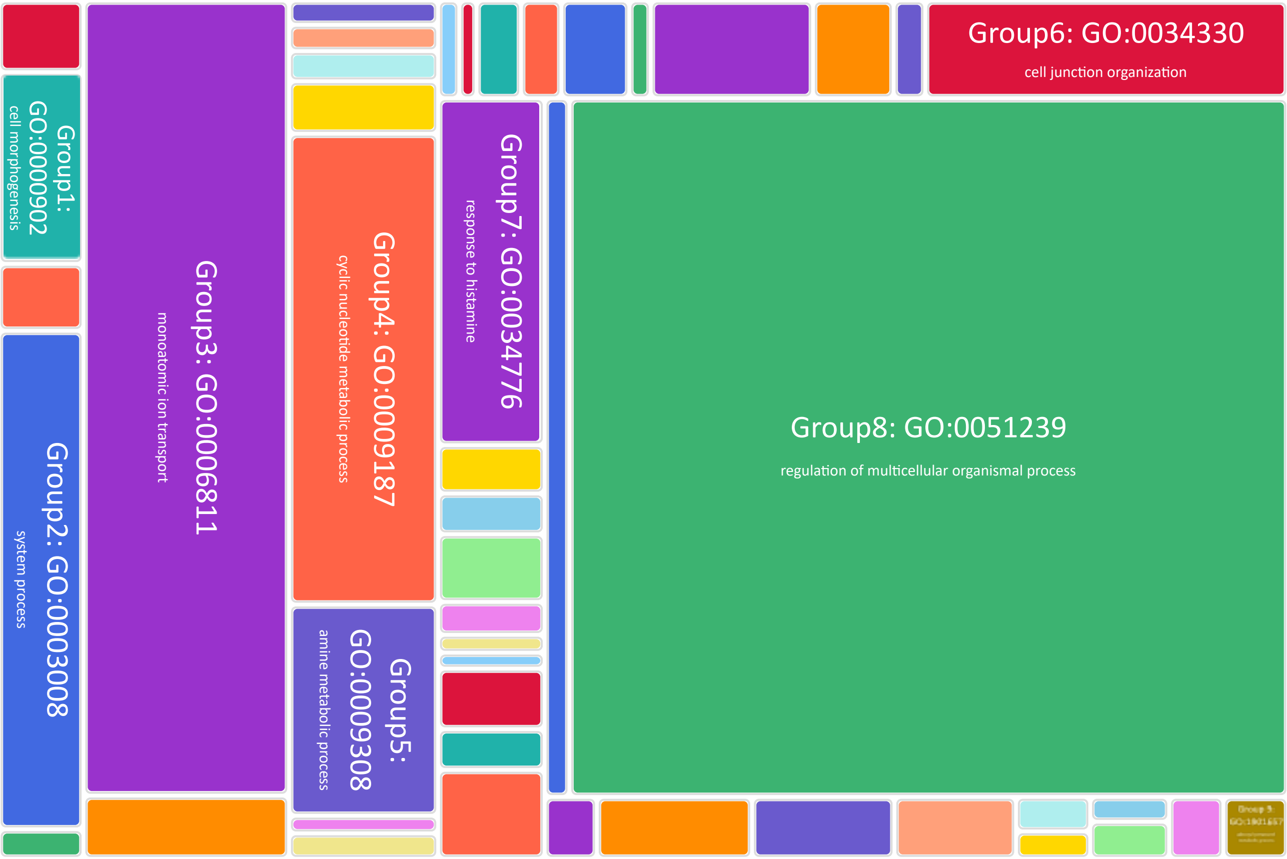

Node 3: *Biomphalaria pfeifferi* GCA_030265305.1
Protein Family Expansion

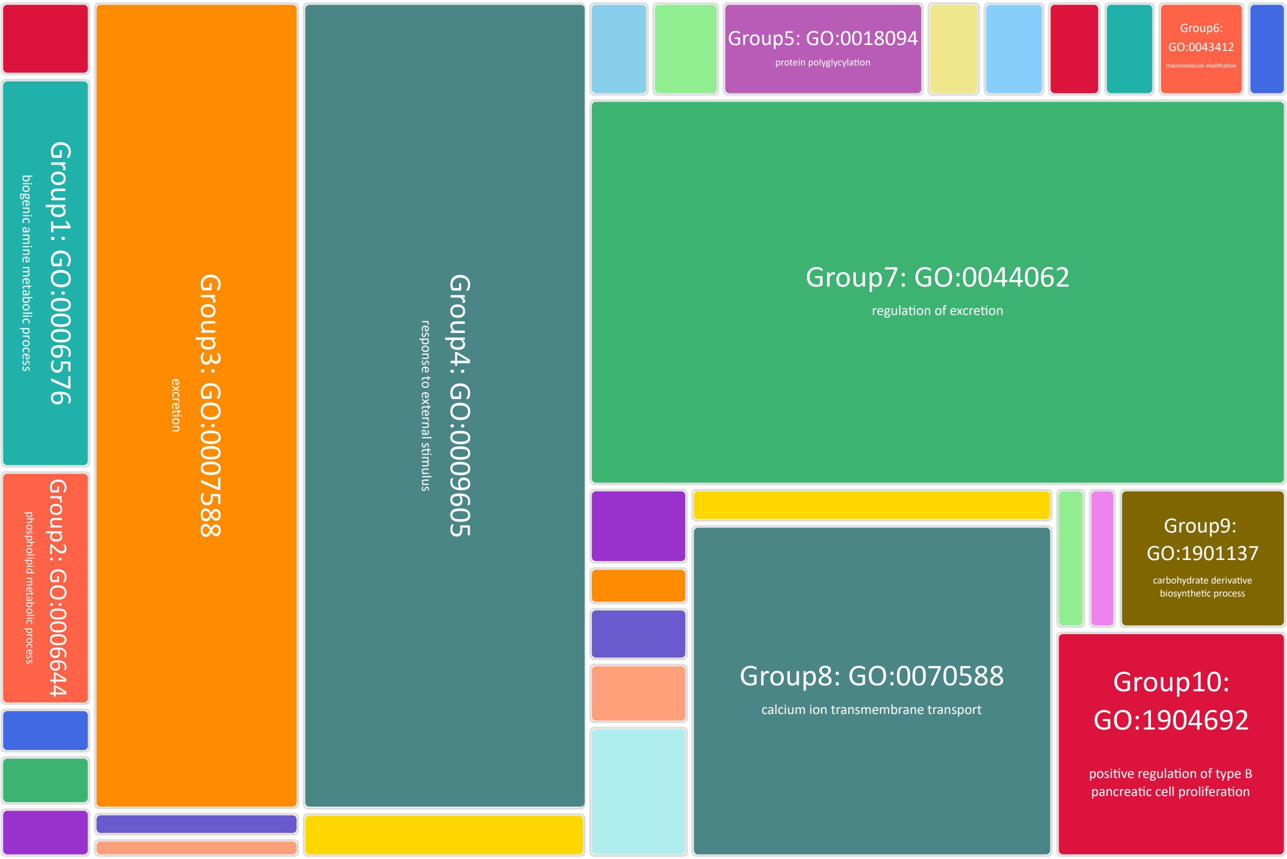

Node 3: *Biomphalaria pfeifferi* GCA_030265305.1
Protein Family Contraction

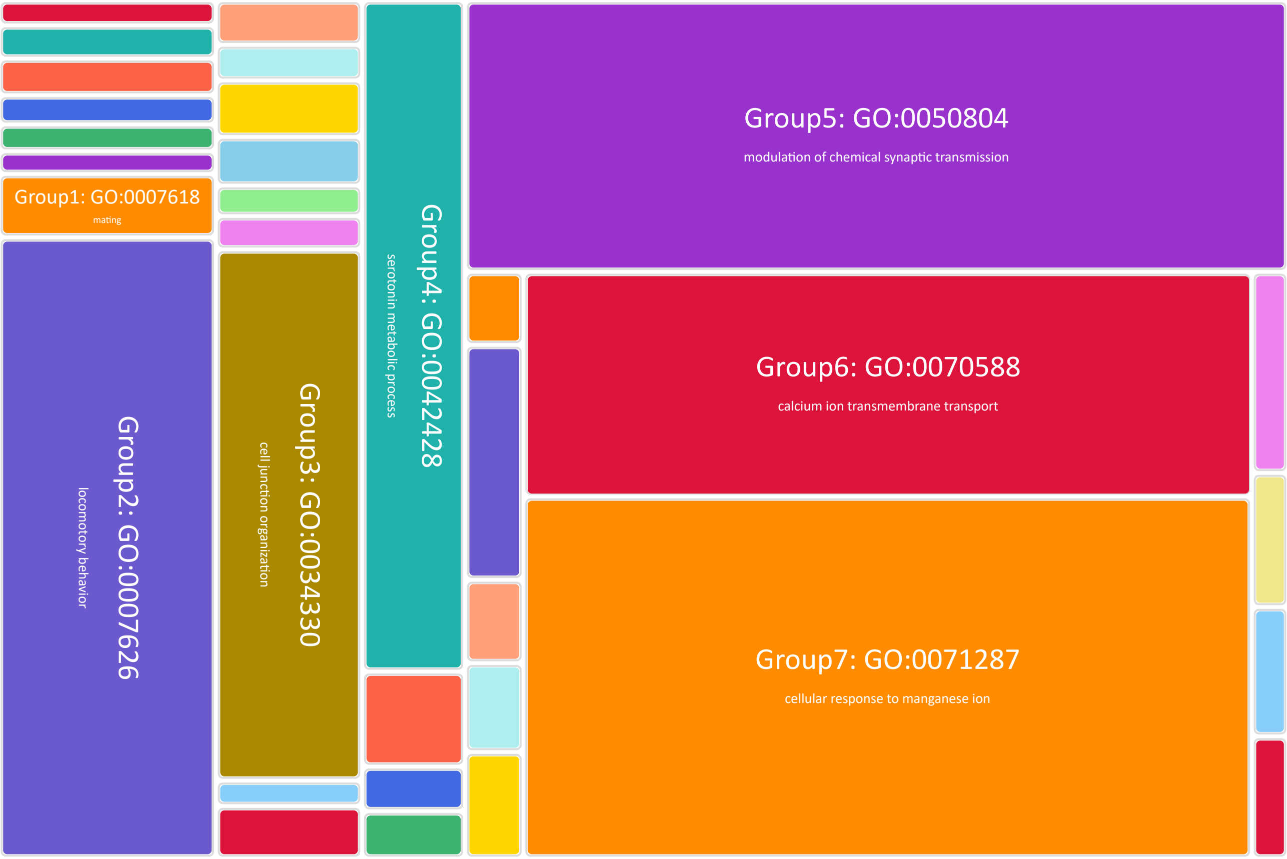

Node 4: *Biomphalaria glabrata* BB02 GCF_000457362.2
Protein Family Expansion

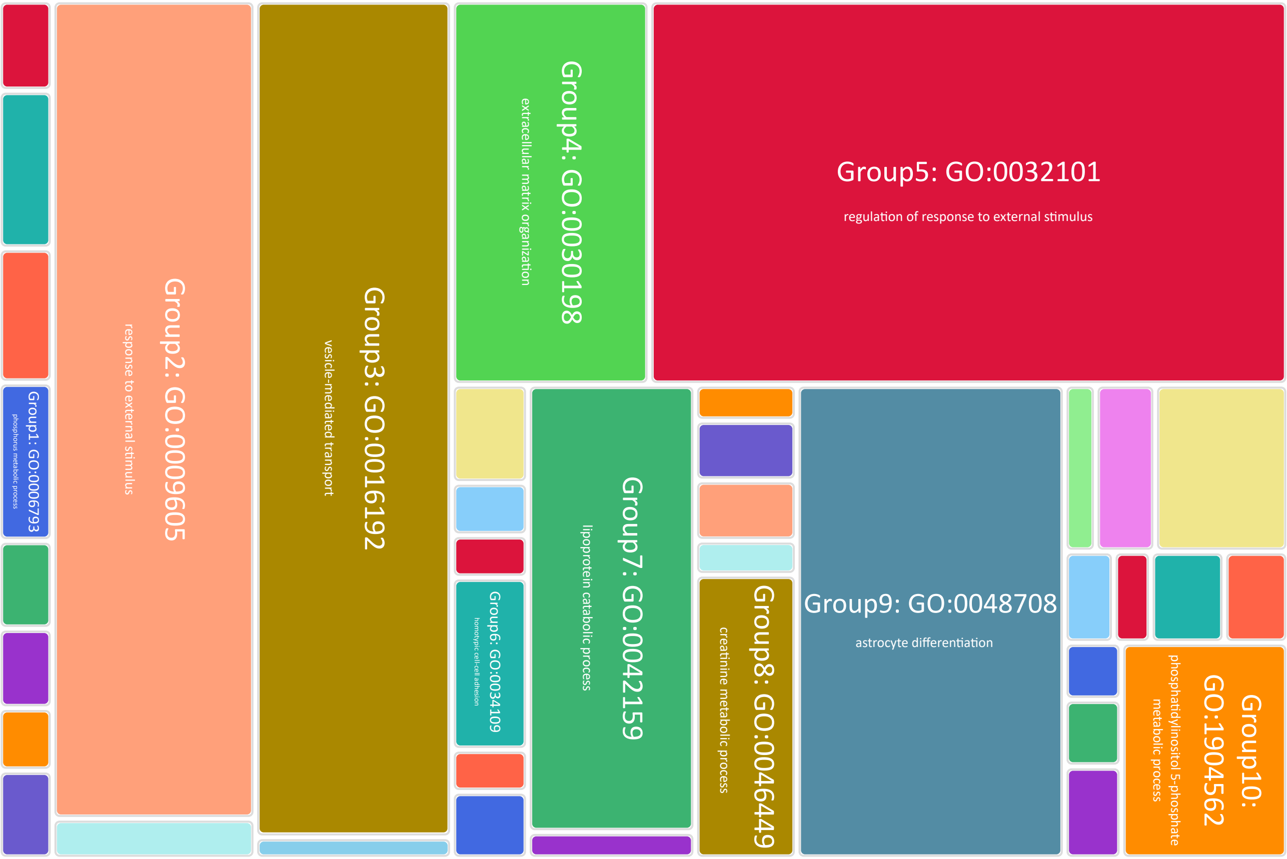

Node 4: *Biomphalaria glabrata* BB02 GCF_000457362.2
Protein Family Contraction

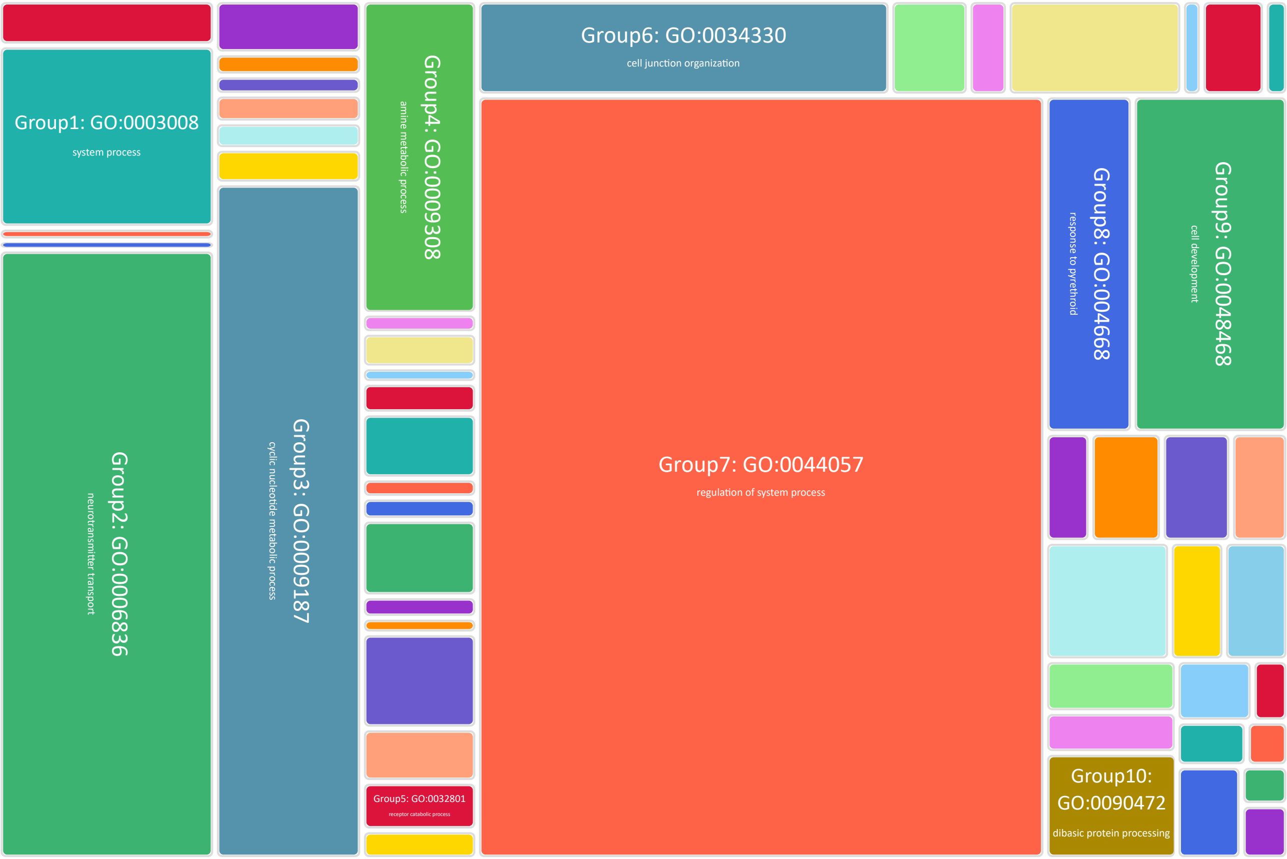

Node 5: *Biomphalaria glabrata* iBS90 x IM ancestor
Protein Family Expansion

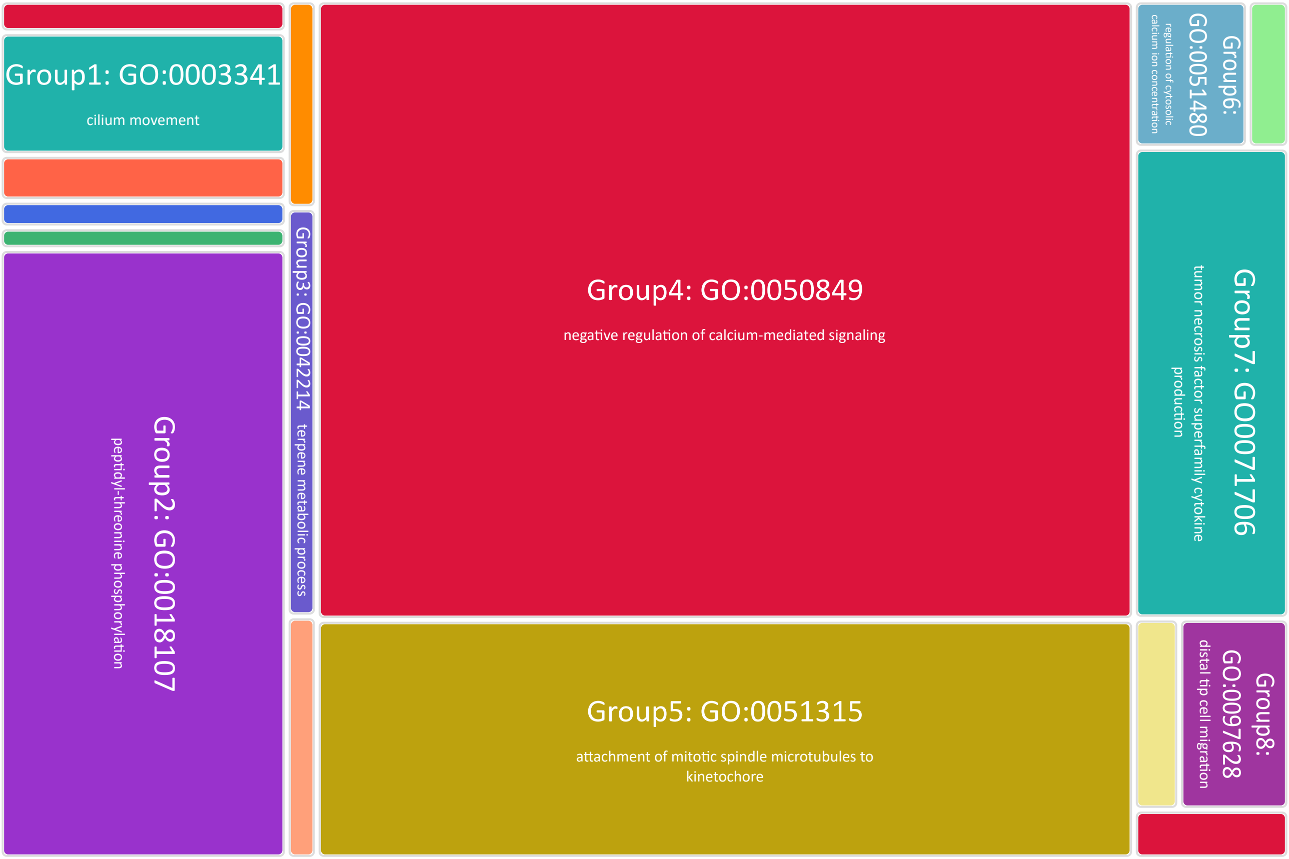

Node 5: *Biomphalaria glabrata* iBS90 x IM ancestor
Protein Family Contraction

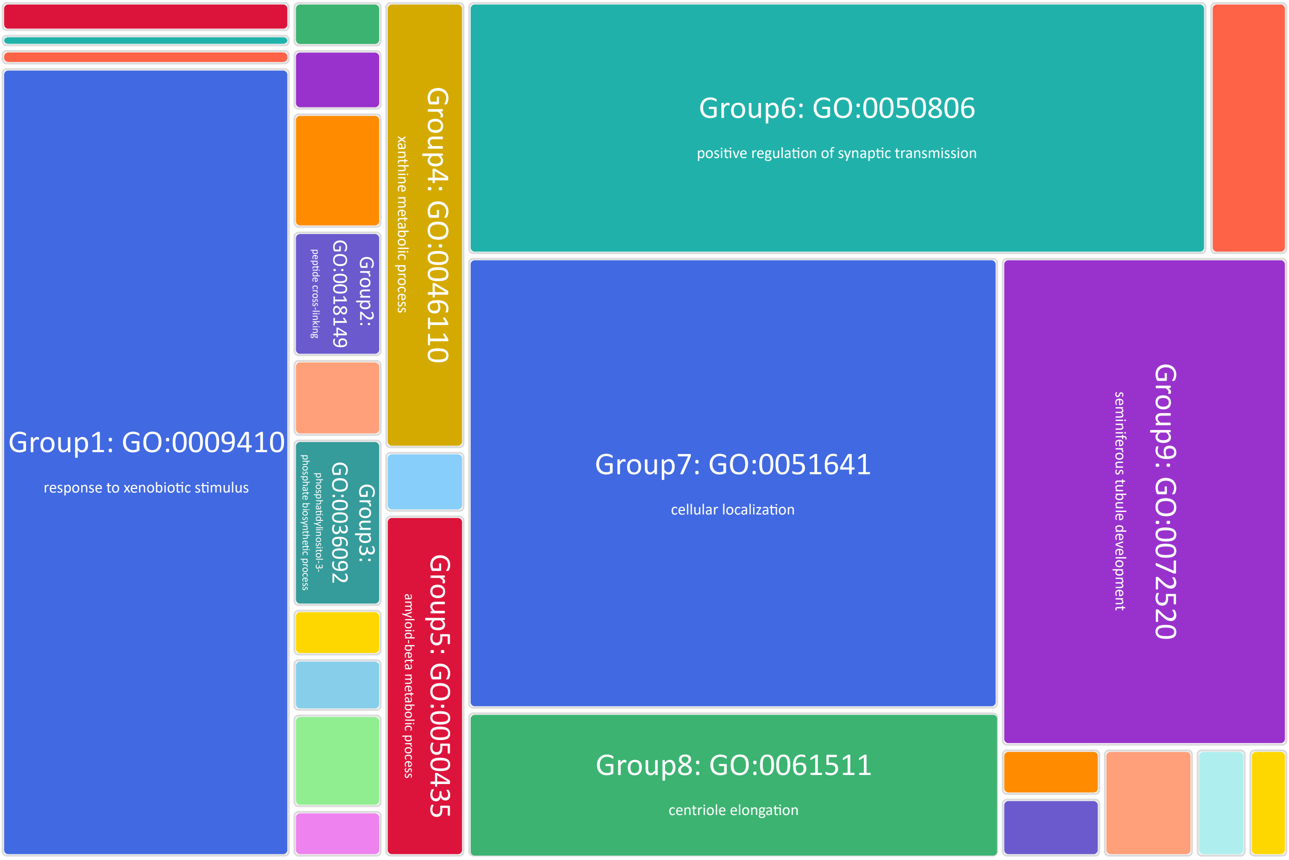

Node 6: African *Biomphalaria sp.*
Protein Family Expansion

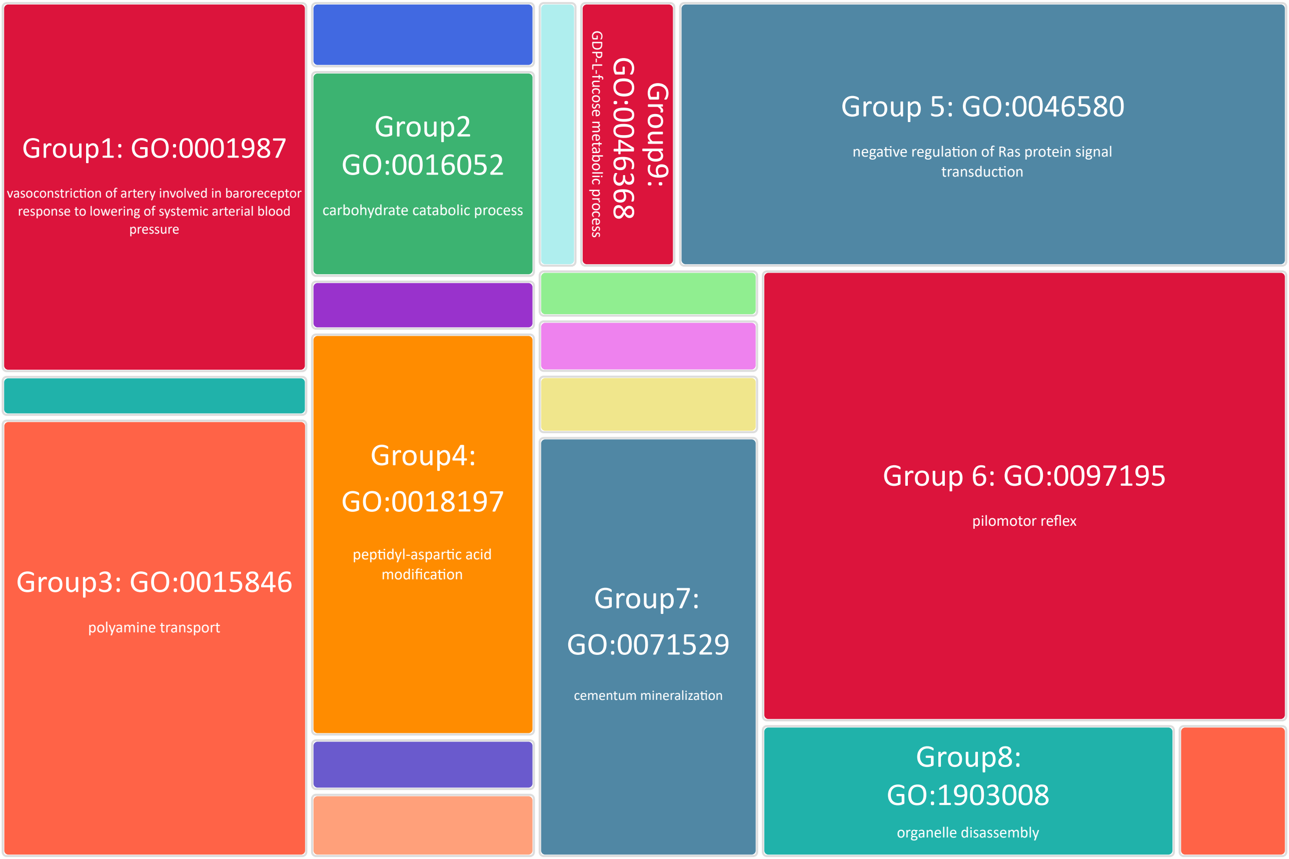

Node 6: African *Biomphalaria sp.*
Protein Family Contraction

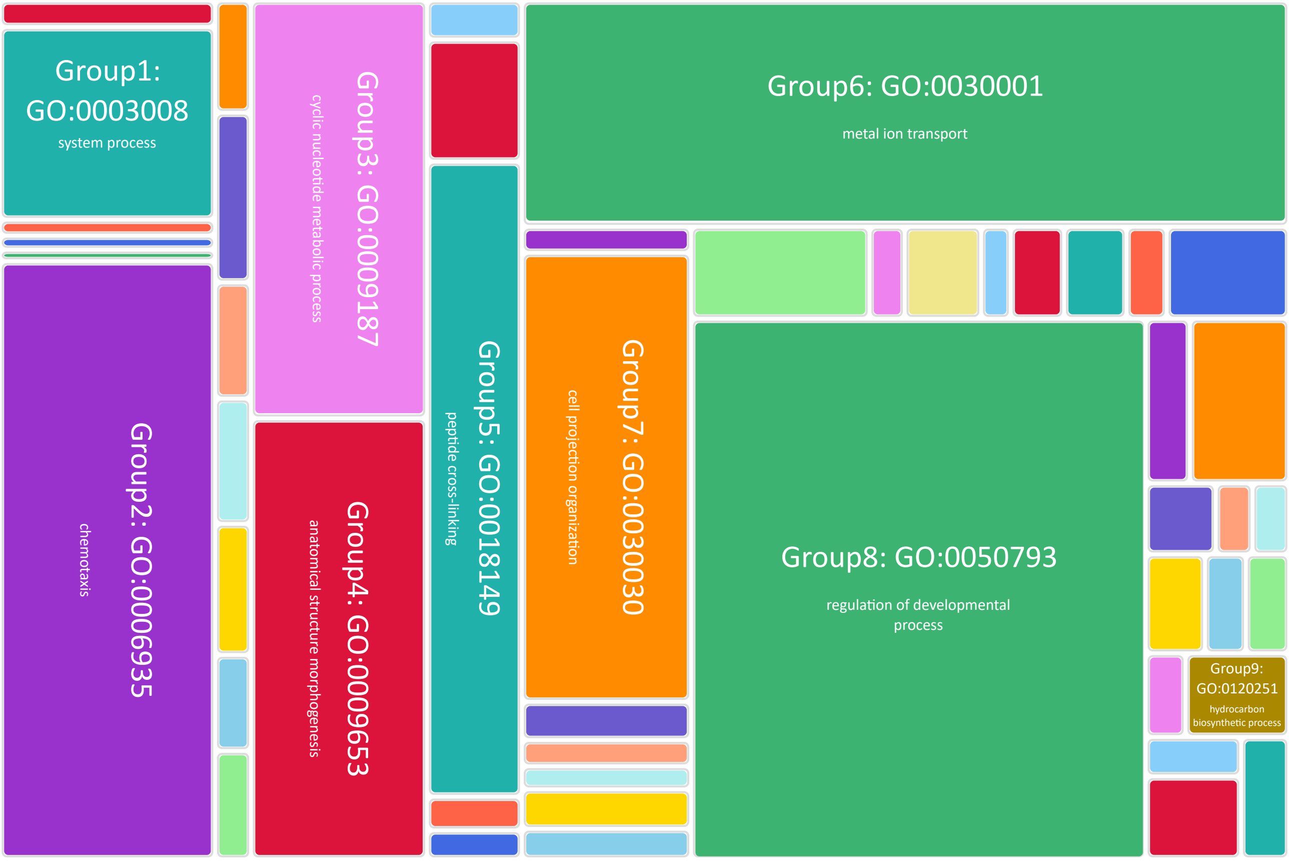

Node 7: *Biomphalaria glabrata* strains ancestor Protein Family Expansion

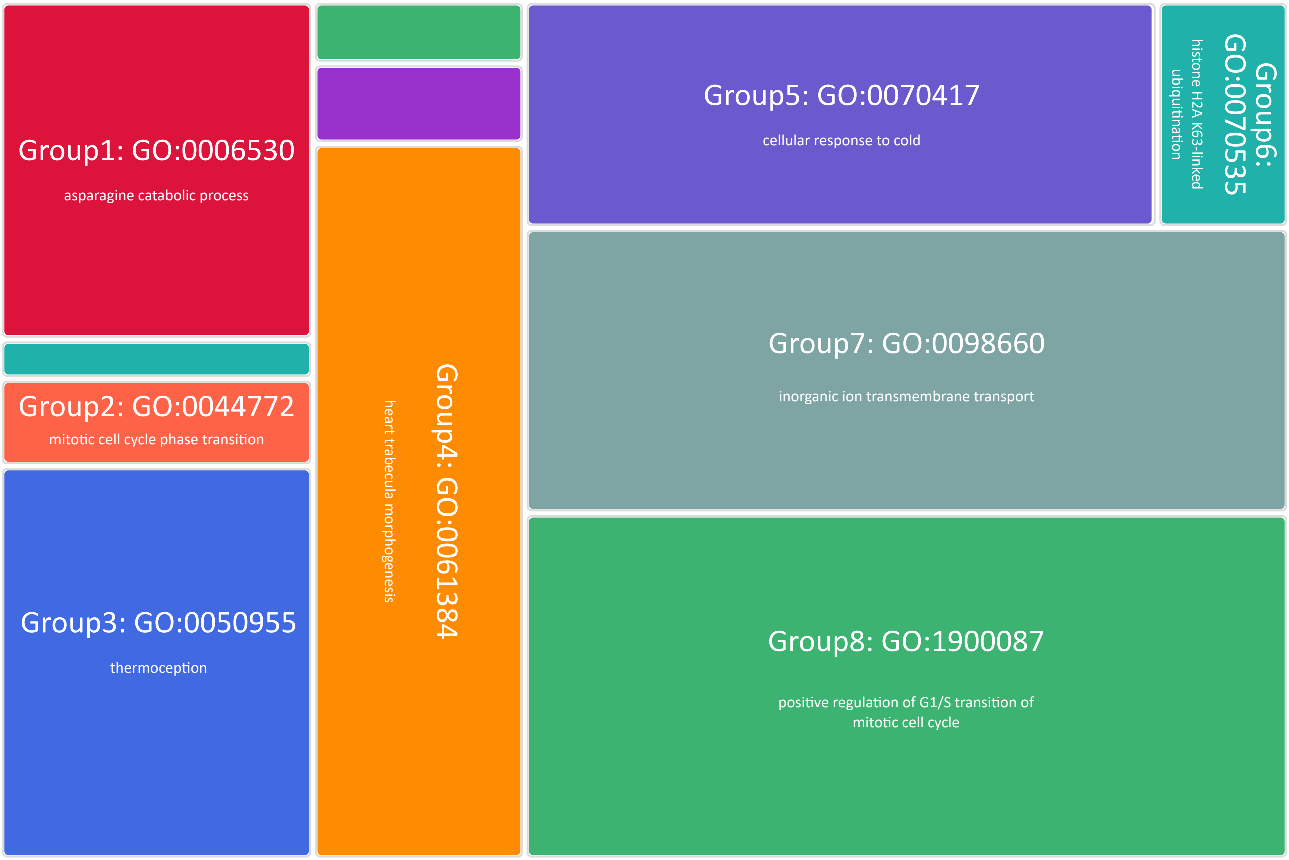

Node 7: *Biomphalaria glabrata* strains ancestor Protein Family Contraction

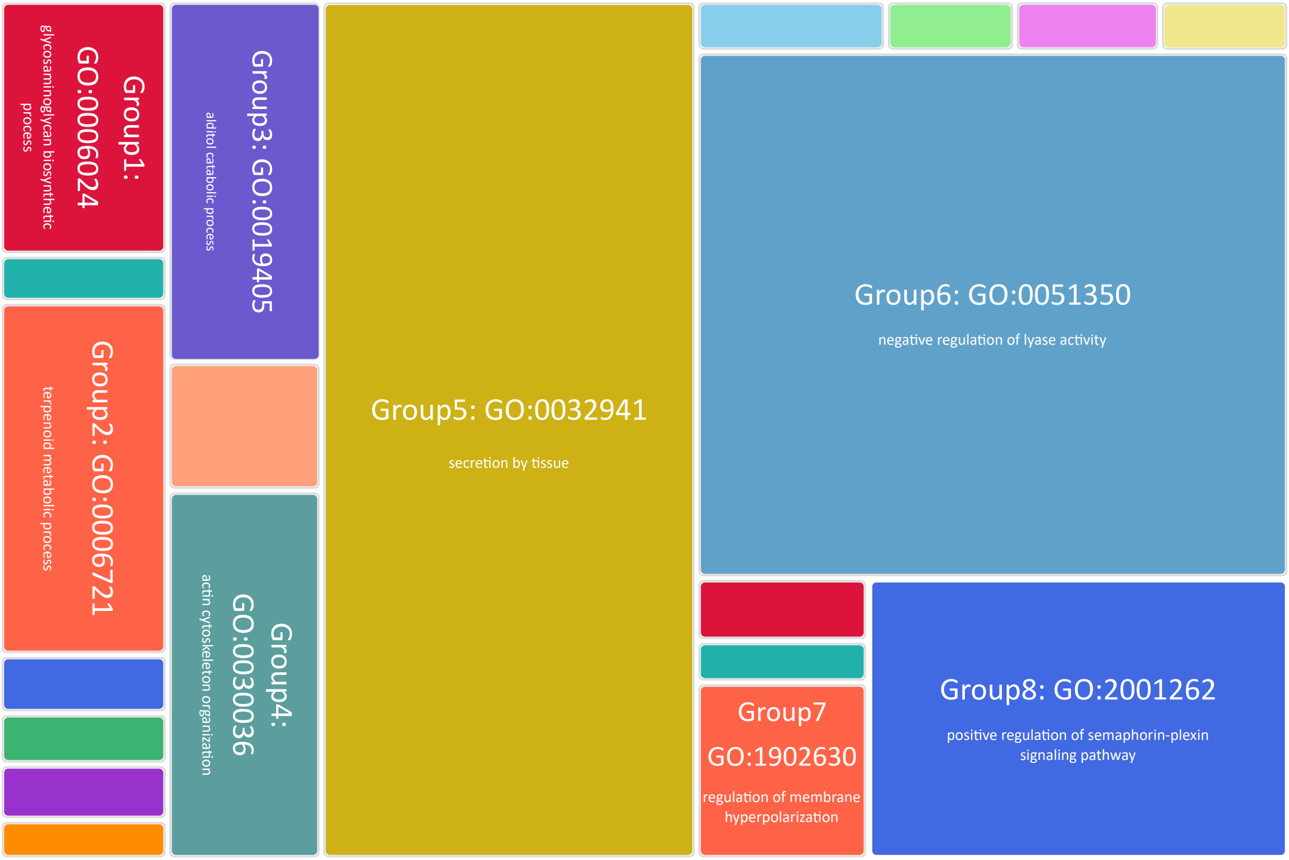

Node 8: *Biomphalaria glabrata* x African species ancestors
Protein Family Expansion

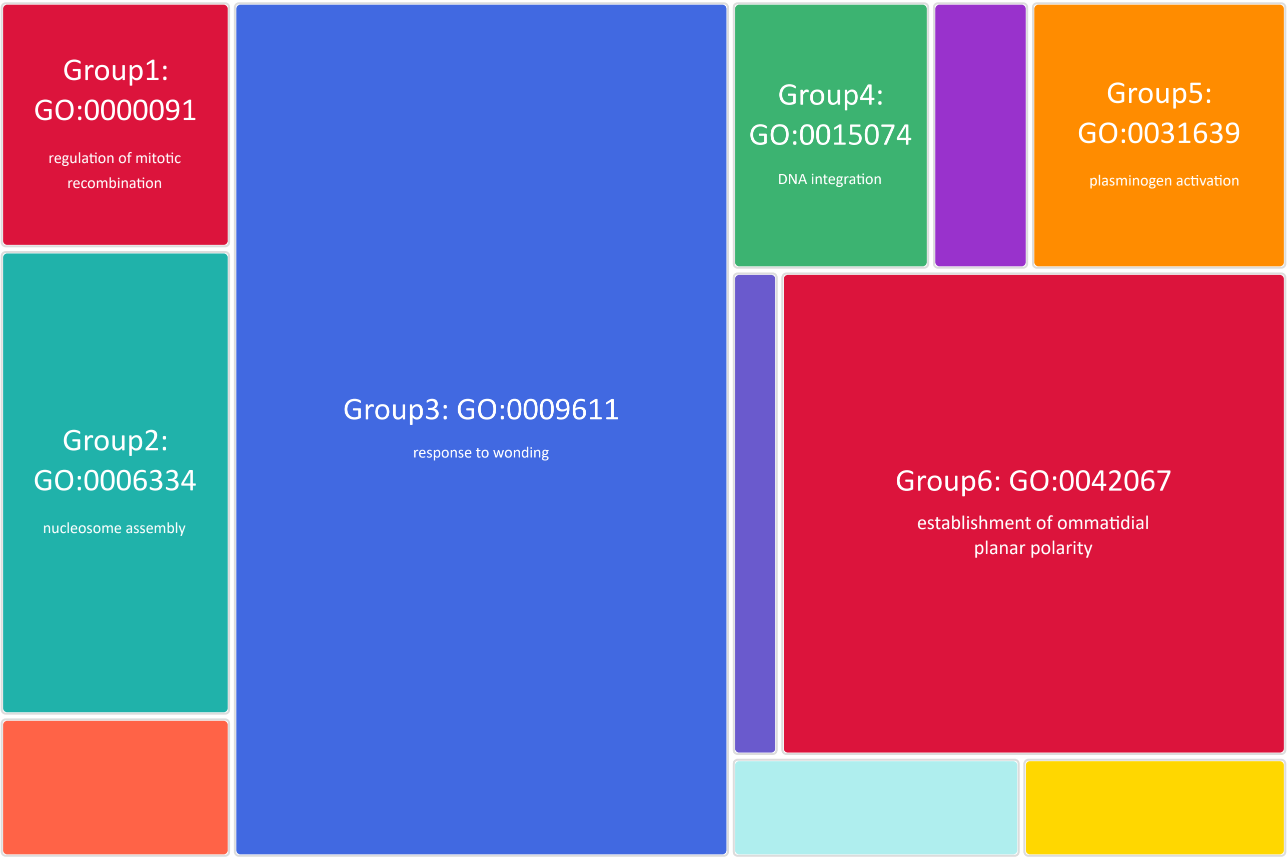

Node 8: *Biomphalaria glabrata* x African species ancestors
Protein Family Contraction

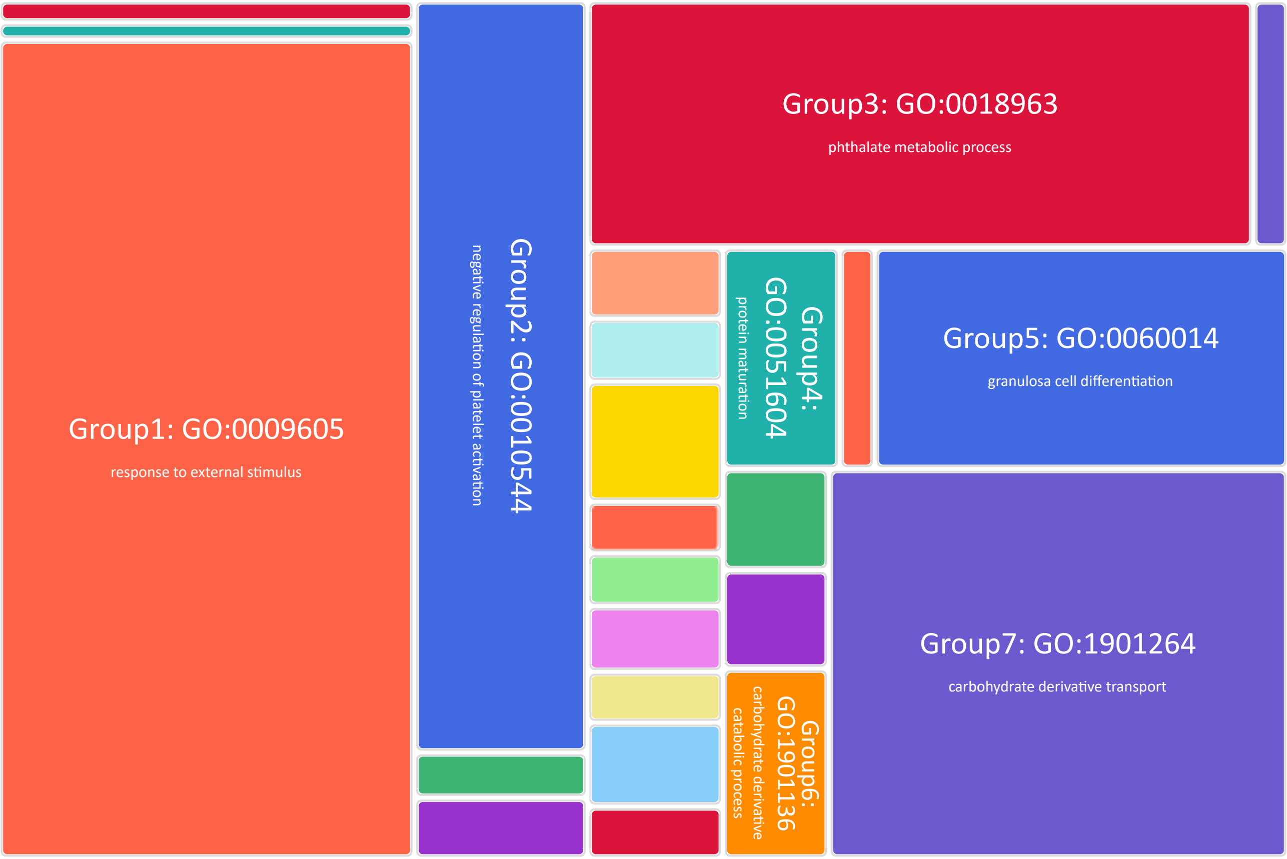

Node 9: *Biomphalaria straminea* GCF_021533235.1
Protein Family Expansion

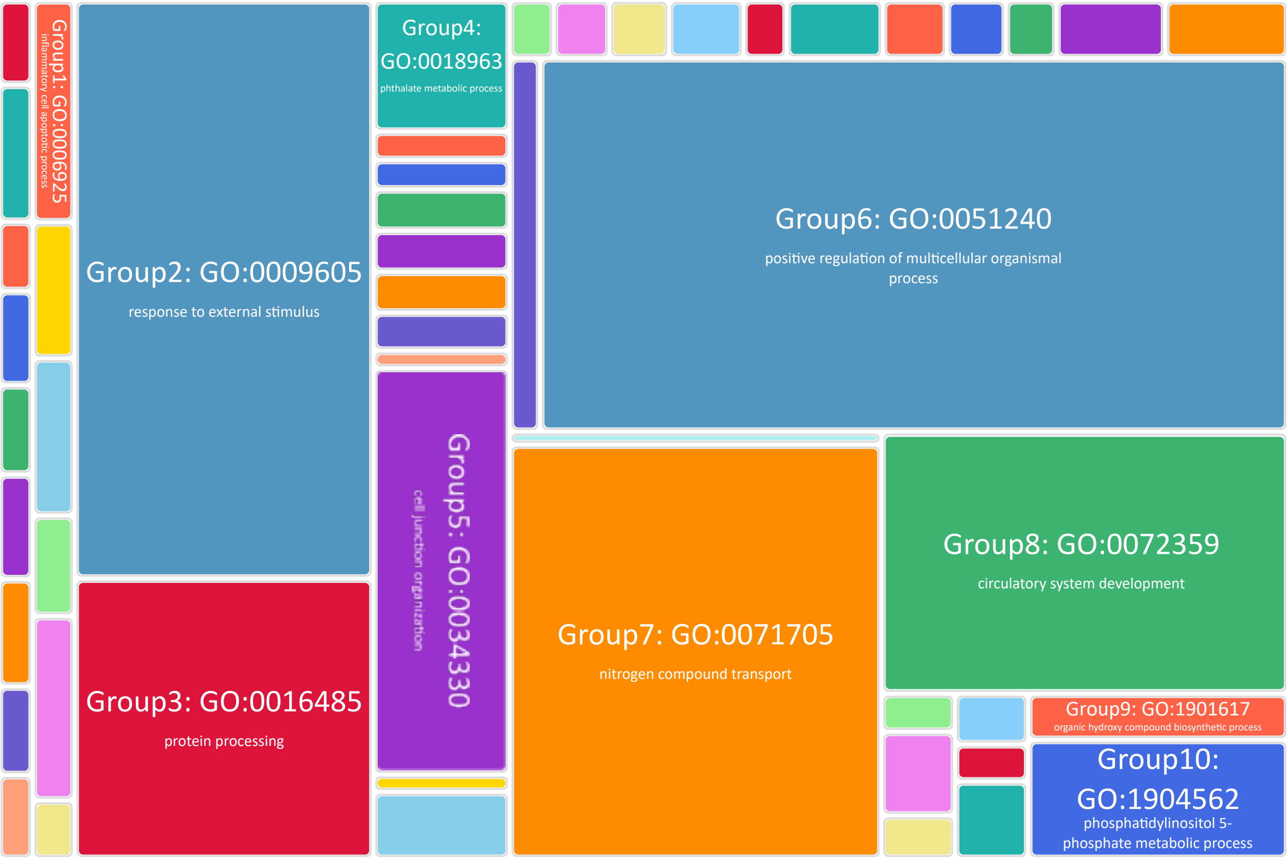

Node 9: *Biomphalaria straminea* GCF_021533235.1
Protein Family Contraction

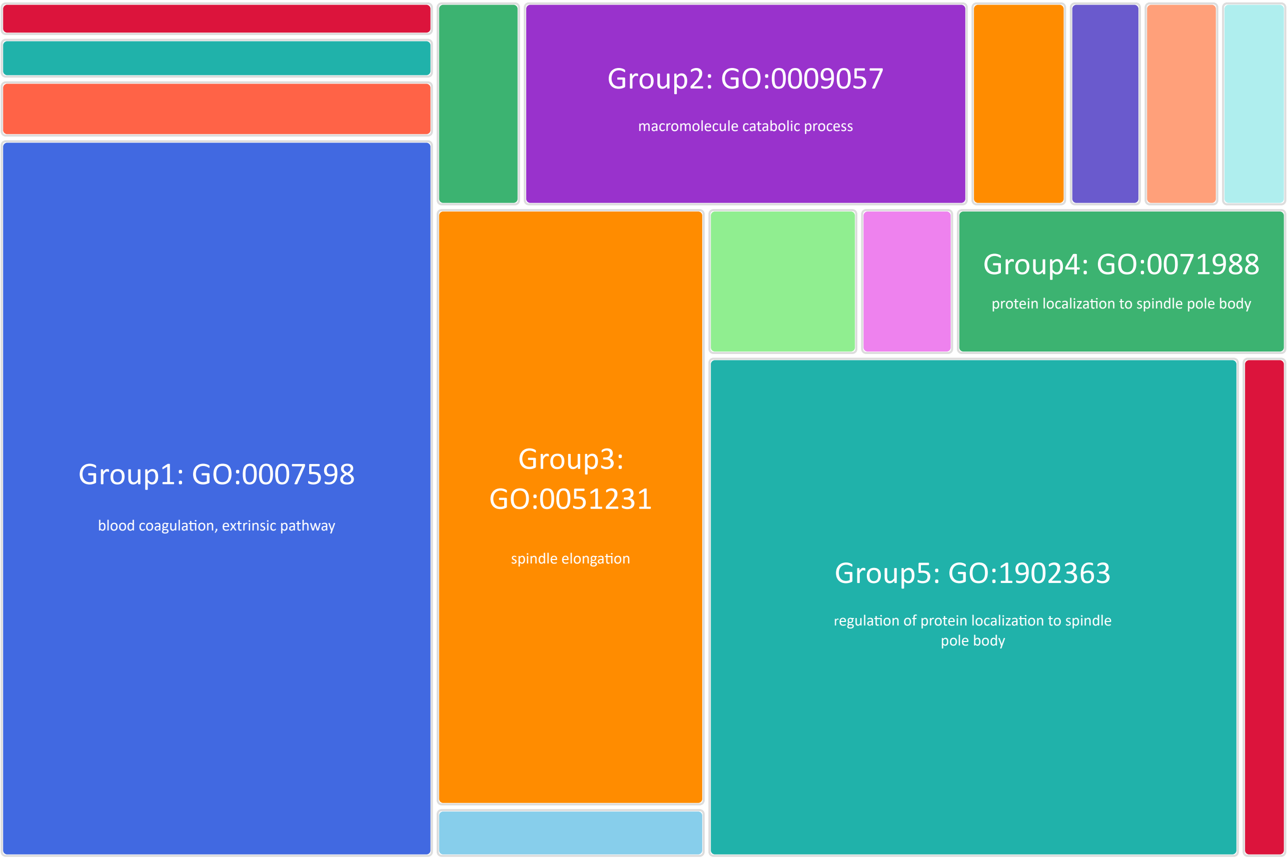

Node 10: Biomphalaria common ancestor
Protein Family Expansion

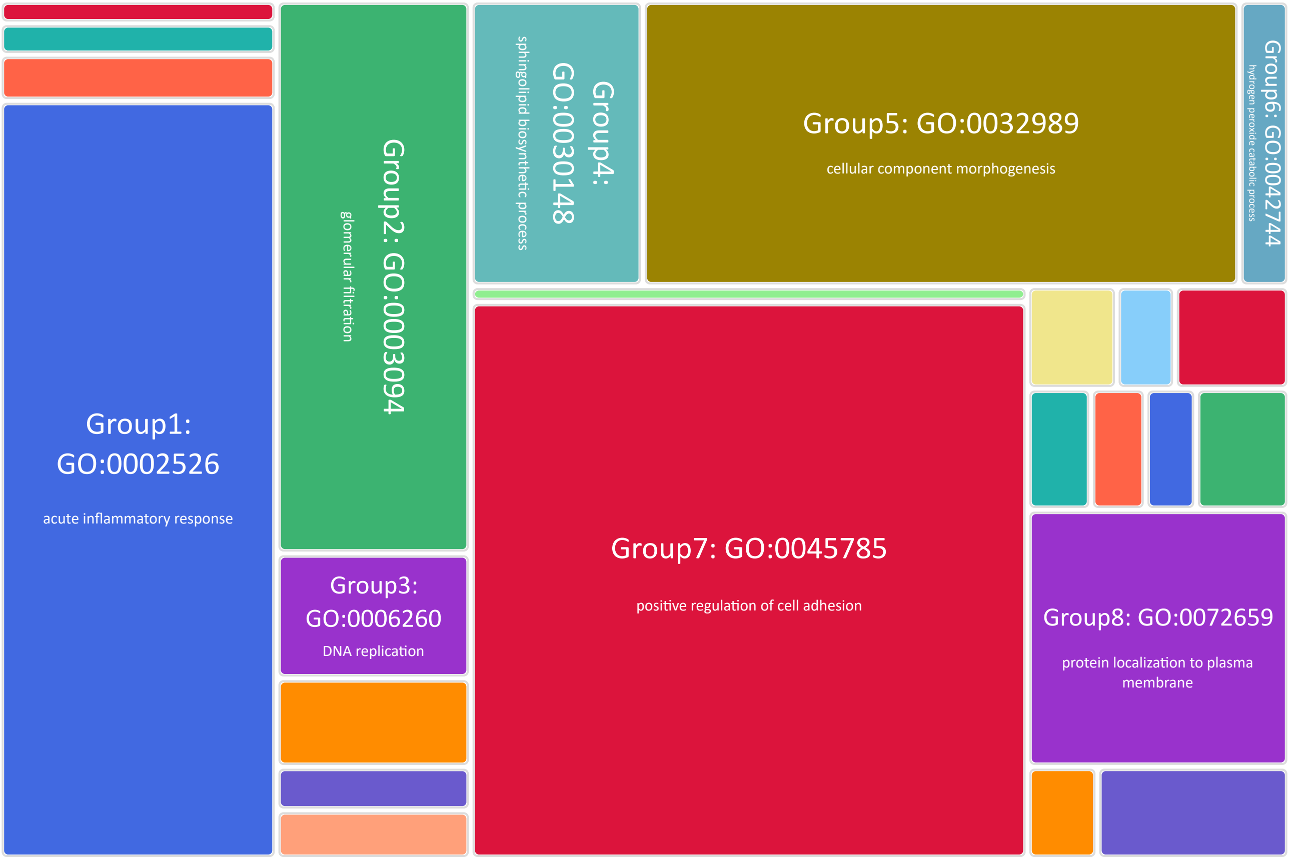

Node 10: Biomphalaria common ancestor
Protein Family Contraction

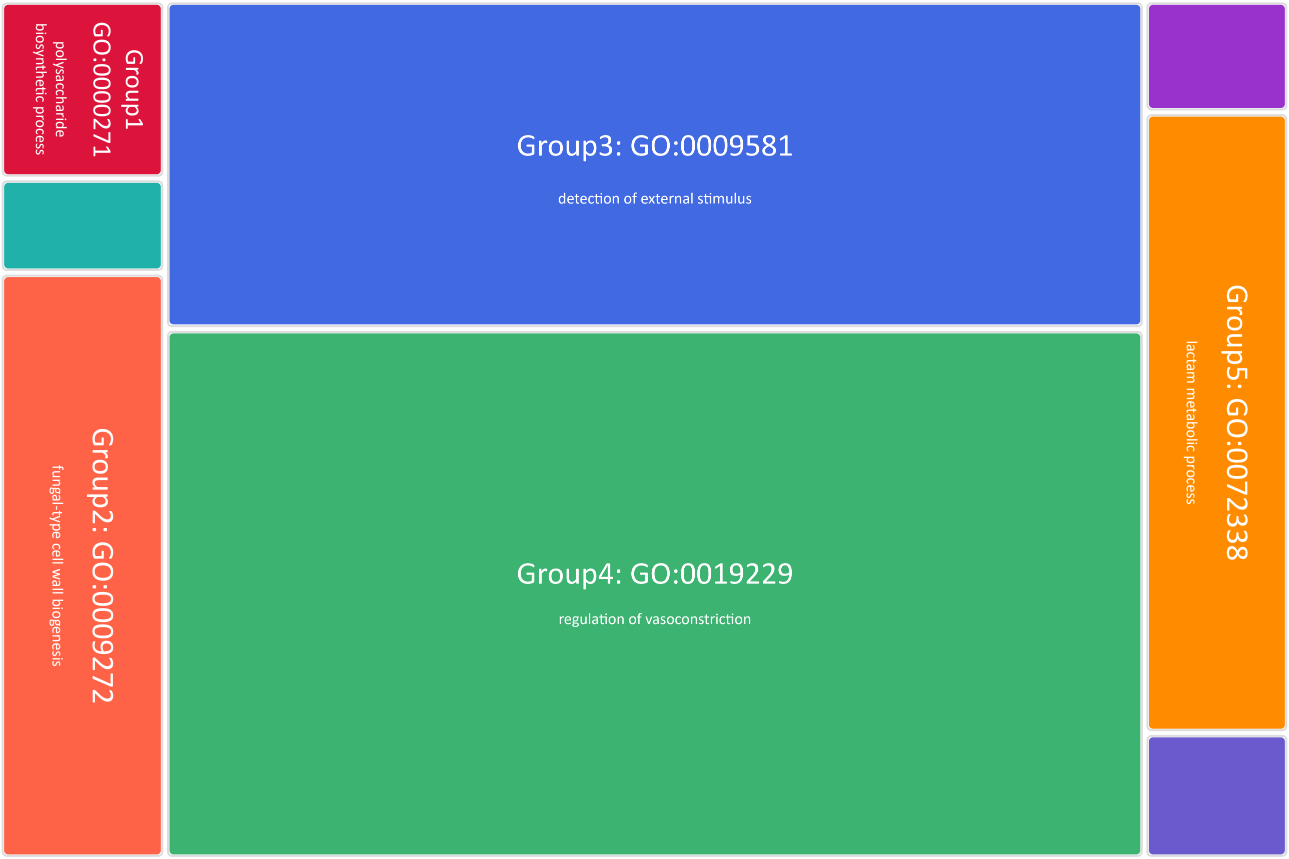

Supplement: Supplementary file 27 — Additional file 27: Supplementary File 5. File providing the REVIGO [74] TreeMap summaries of the GO terms enriched among the protein families, showing significant expansions and contractions for each node (excluding outgroups) of the species tree generated in Orthofinder using the Species Tree of All Genes (STAG) algorithm [68, 75]. [file 12864_2024_10103_MOESM27_ESM.pdf]
